# Tumor Ensemble-Based Modeling and Visualization of Emergent Angiogenic Heterogeneity in Breast Cancer

**DOI:** 10.1038/s41598-019-40888-w

**Published:** 2019-03-27

**Authors:** Spyros K. Stamatelos, Akanksha Bhargava, Eugene Kim, Aleksander S. Popel, Arvind P. Pathak

**Affiliations:** 1 Department of Biomedical Engineering, The Johns Hopkins University School of Medicine, Maryland, USA; 2 Russell H. Morgan Department of Radiology and Radiological Science, The Johns Hopkins University School of Medicine, Maryland, USA; 3 Sidney Kimmel Comprehensive Cancer Center, The Johns Hopkins University School of Medicine, Maryland, USA

## Abstract

There is a critical need for new tools to investigate the spatio-temporal heterogeneity and phenotypic alterations that arise in the tumor microenvironment. However, computational investigations of emergent inter- and intra-tumor angiogenic heterogeneity necessitate 3D microvascular data from ‘whole-tumors’ as well as “ensembles” of tumors. Until recently, technical limitations such as 3D imaging capabilities, computational power and cost precluded the incorporation of whole-tumor microvascular data in computational models. Here, we describe a novel computational approach based on multimodality, 3D whole-tumor imaging data acquired from eight orthotopic breast tumor xenografts (i.e. a tumor ‘ensemble’). We assessed the heterogeneous angiogenic landscape from the microvascular to tumor ensemble scale in terms of vascular morphology, emergent hemodynamics and intravascular oxygenation. We demonstrate how the abnormal organization and hemodynamics of the tumor microvasculature give rise to unique microvascular niches within the tumor and contribute to inter- and intra-tumor heterogeneity. These tumor ensemble-based simulations together with unique data visualization approaches establish the foundation of a novel ‘cancer atlas’ for investigators to develop their own *in silico* systems biology applications. We expect this hybrid image-based modeling framework to be adaptable for the study of other tissues (e.g. brain, heart) and other vasculature-dependent diseases (e.g. stroke, myocardial infarction).

## Introduction

Clinical genotyping has recently demonstrated that subpopulations of cancer cells with unique genomes can exist in different regions or ‘microenvironments’ of a patient’s tumor and that these subpopulations can evolve over time^1,2^. A major driver of inter- and intra-tumor heterogeneity is the underlying heterogeneity of the microvasculature, i.e. the irregular microvascular network and resultant abnormal hemodynamics that also pose a formidable hurdle for developing and delivering effective cancer therapies^3^. Therefore, there is a critical need for new computational tools that enable investigations of tumor vascular heterogeneity and the ‘emergent’ phenotypic alterations that result from changes in the tumor microenvironment^3,4^. However, *in silico* investigations of emergent inter- and intra-tumor angiogenic heterogeneity require that we have access to ‘whole-tumor’ 3D microvascular data as well as such data from ‘ensembles’ of tumors^3,5^. Until recently, technical limitations such as the lack of high-resolution 3D imaging techniques capable of whole-tumor coverage, insufficient computational power and cost presented major hurdles to such systems biology approaches. Here, we present a hybrid image-based modeling framework to demonstrate the feasibility of recapitulating inter- and intra-tumor heterogeneity of the breast cancer microenvironment in terms of vascular morphology, tumor hemodynamics and intravascular oxygenation.

The origins of microvascular heterogeneity in different organs have been investigated in numerous studies using experimental and computational approaches^6–11^. Improvements in imaging resolution now permit acquisition of the 3D microvascular structure and quantification of the morphology of individual microvessel segments within solid tumors^12–14^ and other tissues^15,16^. Moreover, combinations of imaging and mathematical modeling now permit detailed vessel-by-vessel simulations of microvascular blood flow and oxygen transport^15,17,18^. These advances have been accompanied by the development of new computational approaches for modeling hemodynamics, the transport of oxygen and other molecules such as growth factors and nanoparticle-mediated drug transport in realistic tumor vasculatures^19^. However, traditional image-based computational frameworks are not well suited to modeling the emergent phenotypic heterogeneity that arises from the whole-tumor microvasculature because they are limited by the sparse spatial coverage of the underlying tumor microvascular network (usually of the order of <1 mm^3^)^15,17,18^.

To overcome these limitations, we developed an image-based modeling framework and specialized data visualization approaches that allowed us to incorporate 3D, high-resolution (~µm) whole-tumor microvascular images in models of inter- and intra-tumor phenotypic heterogeneity of the breast cancer microenvironment. We leveraged a multimodality imaging pipeline that exploits the strengths of complementary imaging modalities such as *in vivo* MRI, *ex vivo* micro-CT (μCT) and magnetic resonance microscopy (MRM)^20^ to generate an ensemble of high fidelity, high-resolution (~µm) whole-tumor microvascular networks (~GB size dataset) from eight orthotopic breast tumor xenografts. We developed computationally convenient approaches to model hemodynamics and intravascular oxygenation in these whole-tumor microvascular networks. This image-based computational framework made it possible to recapitulate whole-tumor angiogenic heterogeneity in terms of: (i) morphology of the tumor microvascular network; (ii) tumor hemodynamics; and (iii) intravascular oxygenation. We also present novel approaches for visualizing these parameters in 3D space to elucidate the spatial heterogeneity of the angiogenic phenotype within and between whole-tumor microvascular networks. Our tumor ensemble-based analyses of heterogeneity revealed larger dispersion in tumor hemodynamic indices than indices of microvascular morphology. We also discovered that heterogeneity of the angiogenic phenotype led to the emergence of distinct vascular niches within the tumor that uniquely shape its hemodynamic landscape. Finally, we characterized the emergent phenotype arising from these different tumor microenvironments based on their vascular morphology and hemodynamics for the entire tumor ensemble and discovered that hemodynamic parameters alone were insufficient for classifying these different tumor microenvironments. Nevertheless, one could exploit ‘ensemble-based’ metrics of tumor heterogeneity to classify these microenvironments and identify potential new biomarkers.

## Results

### Whole-tumor ensemble derived from 3D multimodality imaging data

**Figure 1:**
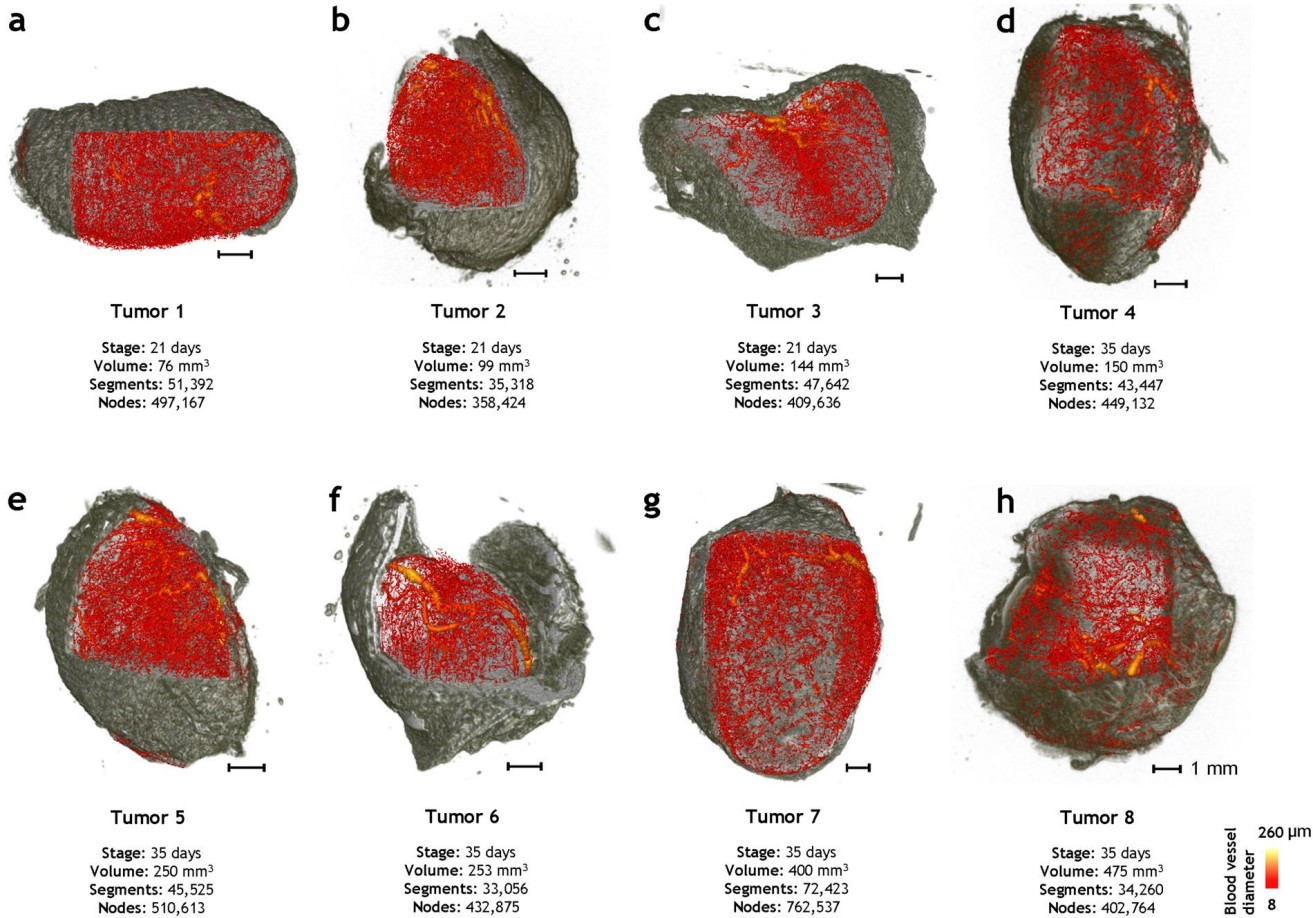
Whole-tumor ensemble derived from 3D multimodality imaging data. (**a**–**c**) Three tumors were excised at 3 weeks post inoculation (i.e. *Day 21* samples), and (**d**–**h**) five tumors were excised five weeks post inoculation (i.e. *Day 35* samples). The soft tissue contrast from *ex-vivo* 3D magnetic resonance microscopy (~40 µm) is rendered in grayscale while the 3D vascular architecture acquired from *ex-vivo* (~8 µm) micro-CT imaging is illustrated with a red color map in which each vessel was scaled and color coded according to its average diameter.

Figure 1 illustrates a 3D whole-tumor ensemble assembled from multimodality images of orthotopic breast tumor xenografts. The soft tissue contrast acquired from magnetic resonance microscopy (MRM) imaging (~40 µm resolution) is shown in grey and the 3D microvascular network acquired from micro-CT (μCT) imaging (8 µm resolution) is shown in a red color map scaled by the mean vessel diameter. Each 3D whole-tumor microvascular network (~GB size dataset) comprised of tens of thousands of vessel segments and a few hundred thousand nodes. The whole-tumor samples are displayed according to the post-inoculation age they were imaged at (i.e. *Day 21* or *Day 35*) wherein the tumor volumes ranged from 76–450 mm^3^. These unique 3D visualizations provide direct insight into the 3D heterogeneity of the angioarchitectural landscape within and between whole-tumors.

### Morphological heterogeneity of whole-tumor microvascular networks

**Figure 2:**
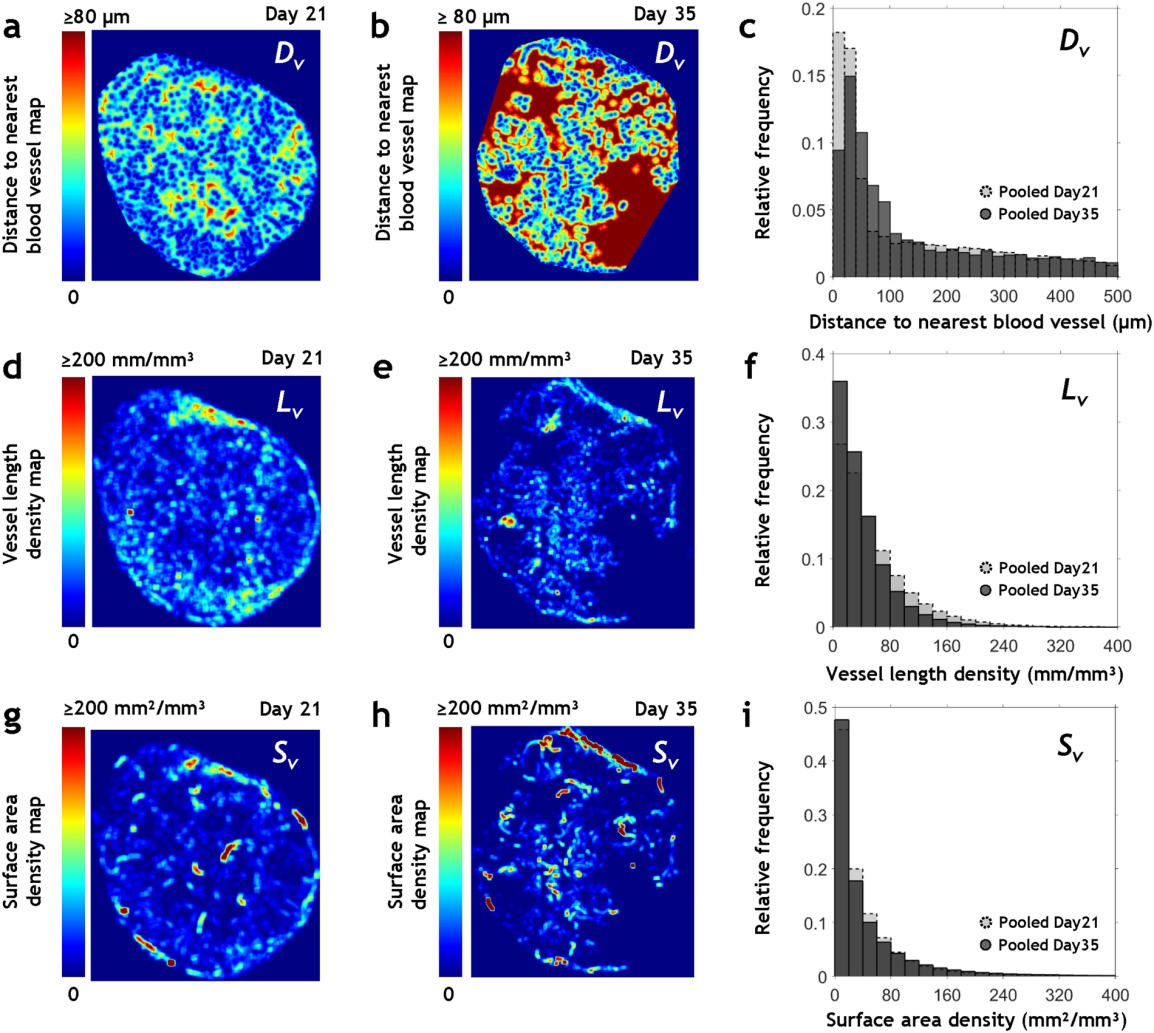
Morphological heterogeneity of whole-tumor microvascular networks. Quantitative visualizations of spatial heterogeneity in: (**a**–**c**) *D*_*v*_; (**d**–**f**) *L*_*v*_, and (**g**–**i**) *S*_*v*_ in whole-tumor microvascular networks. The maximum intensity projection image from a 400 µm thick section from the tumor center was utilized for visualizing the spatial distribution of these parameters in Tumor 1 (smallest *Day 21* sample) in (**a**,**d**,**g**) and for Tumor 8 (largest *Day 35* sample) in (**b**,**e**,**h**), respectively. *D*_*v*_ maps (**a**,**b**) were computed on an 8 µm × 8 µm × 8 µm grid and *L*_*v*_ and *S*_*v*_ maps (**d**,**e**,**g**,**h**) were computed on a 160 µm × 160 µm × 160 µm grid. Probability distribution functions based on pooled *D*_*v*_, *L*_*v*_ and *S*_*v*_ data are shown for *Day 21* tumors (light grey) and *Day 35* tumors (dark grey) in (**c**,**f**,**i**).

Figure 2 shows 2D, high-resolution maps of vascular morphological heterogeneity in *Day 21* and *Day 35* tumors in terms of: distance to nearest vessel (*D*_*v*_), vascular length density (*L*_*v*_) and vascular surface area density (*S*_*v*_), respectively. These quantitative maps illustrate the spatial heterogeneity of tumor vascular morphology and help identify unique regions within the tumor. For example, large avascular areas (i.e. areas exhibiting high *D*_*v*_, and low *L*_*v*_, *S*_*v*_) were visible in the center of a *Day 35* tumor (Fig. 2b,e,h), and areas exhibiting low *D*_*v*_ (Fig. 2a,b**)** and high *L*_*v*_ and *S*_*v*_ (Fig. 2d,e,g,h) were visible in the rims of *Day 35* and *Day 21* tumors. The volume density (i.e. the ratio of vascular volume to tumor volume) decreased with increase in tumor volume - from 0.86 in the smallest *Day 21* tumor (76 mm^3^) to 0.36 in the largest *Day 35* tumor (475 mm^3^). Figure 2c,f,i are the probability distributions of these morphological parameters pooled by post-inoculation age and based on whole-tumor spatial coverage. *Day 21* tumors had a greater percentage of small *D*_*v*_ values (10–60 µm) than advanced *Day 35* tumors. The *Day 21* group also exhibited a larger percentage of high *L*_*v*_ values (100–240 mm/mm^3^) and a higher median *L*_*v*_ of 40.7 mm/mm^3^ in contrast to 30 mm/mm^3^ for the *Day 35* group. Pooled median *S*_*v*_ varied from 23.2 mm²/mm³ in *Day 21* tumors to 22 mm²/mm³ in *Day 35* tumors. Correspondingly, there were a greater number of dilated vessels in *Day 35* tumors as indicated by the pooled median diameter of 13 µm versus 11 µm for *Day 21* tumors.

### Predictions from image-based hemodynamic modeling match those from conventional modeling approaches

We implemented our blood transport model for a rat mesentery microvascular network with boundary conditions based on experimental *in vivo* data as described in Pries *et al*.^21^. We observed excellent agreement between our simulated data and that from Pries *et al*.^21^, as indicated by R^2^ = 0.99 and 0.93 for blood flow rates and discharge hematocrit, respectively (Supplementary Fig. S1a,b). Next, we compared the results of hemodynamic simulations based on our tumor ensemble data with experimental and simulated data from the extant literature. Supplementary Tables S1 and S2 summarize the mean morphological and functional parameters computed for the tumor ensemble along with the corresponding ranges (i.e. mean ± standard deviation) derived from the literature^22–33^. The literature data exhibited large variations in morphological and functional parameters due to the varying sizes and tumor types used in these xenograft studies. Wherever possible, we compared our values against data for similar tumor type and size. As shown in Supplementary Tables S1 and S2, the mean values for our morphological and simulated hemodynamic parameters were in good agreement with the ranges reported in the literature.

### Visualizing the spatial heterogeneity of whole-tumor hemodynamics and intravascular oxygenation

**Figure 3:**
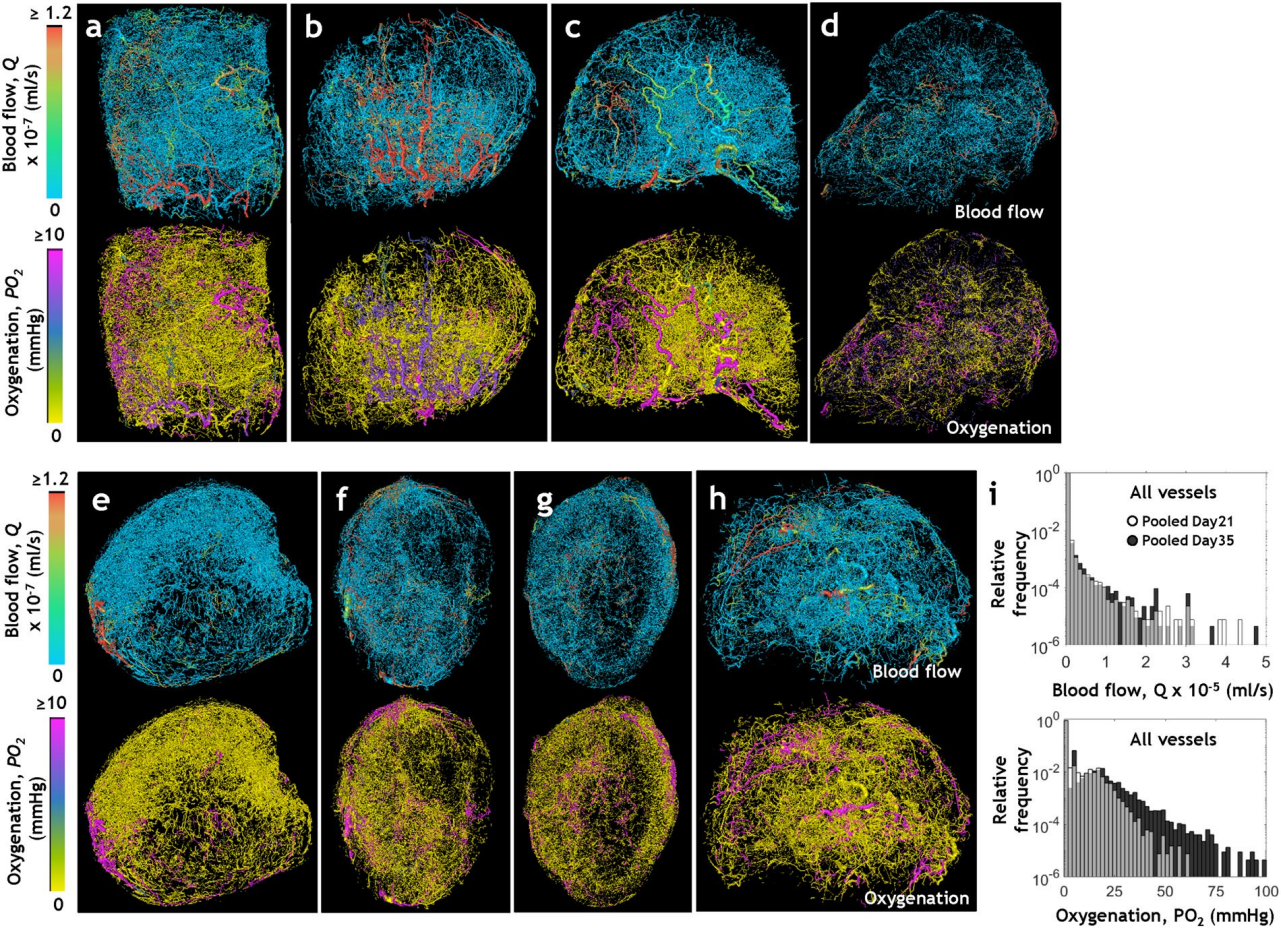
Visualizing the spatial heterogeneity of whole-tumor hemodynamics and intravascular oxygenation. 3D visualizations of hemodynamic heterogeneity in microvascular networks derived from (**a**–**c**) *Day 21* tumors and (**d**–**h**) *Day 35* tumors wherein the vessels are color coded by their simulated blood flow rates, *Q* × 10^−7^ (ml/s) (in top row) and intravascular oxygenation, *PO*_*2*_ values (mm Hg) (in bottom row). (**i**) Pooled frequency distributions of simulated *Q* (top row) and *PO*_*2*_ (bottom row) for all vessels of *Day 21* tumors (light grey) and *Day 35* tumors (dark grey).

Figure 3 illustrates the spatial heterogeneity of the blood flow rate, *Q* (ml/s) and intravascular oxygenation *PO*_*2*_ (mmHg) computed for each whole-tumor microvascular network. We observed well-perfused and well-oxygenated vessels within the tumor rim of *Day 21* samples (Fig. 3a–c) whereas such vessels were unevenly distributed within the tumor volume of *Day 35* samples (Fig. 3d–h). Pooled hemodynamic data from 133,036 vessels from *Day 21* tumors, and 226,552 vessels from *Day 35* tumors are plotted in Fig. 3i. The upper panel shows the distribution of blood flow rate and the bottom panel that of intravascular oxygenation. The distributions of blood flow rate and intravascular oxygenation were positively skewed, indicating that a large sub-population of tumor vessels (~90%), in both *Day 21* and *Day 35* tumors, were minimally or poorly perfused (0 ≤ *Q* ≤ 2.5 × 10^−3^ µl/s) as well as under-oxygenated (0 ≤ *PO*_*2*_ ≤ 5 mmHg). This was also evident from the visualizations shown in Fig. 3a–h. A small fraction of *Day 35* tumor vessels (0.5% in tumors 7 and 8, respectively) exhibited high oxygenation values (60–87 mmHg), which resulted in the *Day 35 PO*_*2*_ distribution having a longer tail than the *Day 21* distribution.

**Figure 4:**
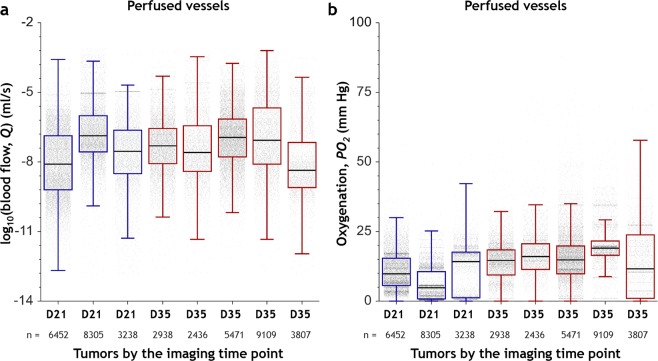
Inter- and intra-tumor hemodynamic heterogeneity of perfused regions. Distributions of (**a**) *Q* (ml/s) and (**b**) *PO*_*2*_ (mm Hg) using data from all the perfused vessels (i.e. *Q* ≥ 10^−10^ µl/s) in three D21 (*Day 21*) tumors and five D35 (*Day 35*) tumors. The box and whisker plots of these distributions show the median, interquartile range (IQR) and the data within the Q1 − 1.5IQR and Q3 + 1.5IQR range. Tumors are arranged along the x-axis in the order of increasing tumor volume. The number of perfused vessel segments (*n*) for each tumor is indicated on the secondary x-axis.

Analysis of blood flow rate and intravascular oxygenation heterogeneity in perfused regions of whole-tumors, i.e. regions not devoid of blood flow (*Q* ≥ 10^−10^ µl/s) demonstrated profound inter- and intra-tumor hemodynamic heterogeneity (Fig. 4a,b). Collectively, these data clearly demonstrate the inter- and intra-tumor heterogeneity of perfusion and oxygenation that arises from the heterogeneous microvascular morphologies of these tumors.

### Emergent whole-tumor hemodynamics were more heterogeneous than their underlying microvascular morphology

**Figure 5:**
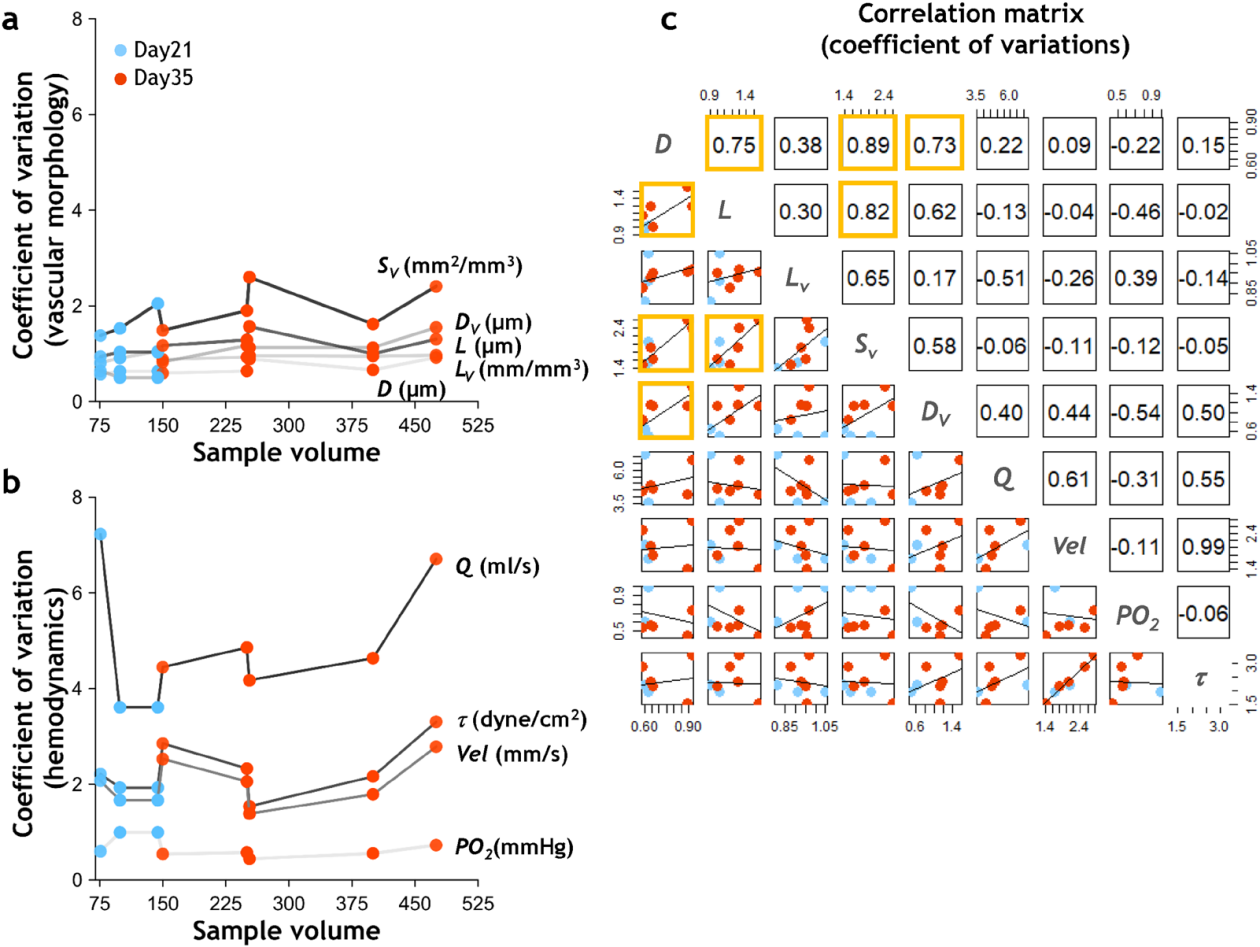
Emergent whole-tumor hemodynamics were more heterogeneous than their underlying microvascular morphology. *CV* of (**a**) morphological parameters - *D*, *L*, *L*_*v*_, *S*_*v*_, and *D*_*v*_ - based on data from all vessels within each tumor; and (**b**) hemodynamic parameters - *Q*, *Vel*, *τ*, and *PO*_*2*_ - based on data from all the perfused vessels within each tumor. Tumors are arranged along the x-axis in the order of increasing tumor volume. Blue dots represent *Day 21* tumors and orange dots represent *Day 35* tumors. (**c**) Pairwise Pearson correlation matrix between morphological *CV*s and hemodynamic *CV*s. The upper triangular matrix illustrates the correlation coefficients, while the lower triangular matrix exhibits the underlying data from (**a**,**b**) and the linear fit to these values. Statistically significant entries (*p* < 0.05) are highlighted by orange boxes.

Figure 5a,b show the coefficient of variation (*CV*) for each tumor across different vascular parameters. These plots indicate that the distribution of blood flow rate (Fig. 5b) was more heterogeneous than those for the underlying vascular morphological parameters (Fig. 5a). Moreover, the dispersion in blood flow was larger than the dispersion in intravascular oxygen tension by more than two orders of magnitude. Vascular parameters, e.g. diameter *D*, length *L*, length density *L*_*v*_, distance to nearest vessel *D*_*v*_ and surface area density *S*_*v*_ exhibited *CV* values ≤ 2.5, whereas functional parameters e.g. blood flow rate (*Q*), shear stress (*τ*), and velocity (*Vel*) exhibited *CV* values ranging from 2–8, i.e. were more heterogeneous. Moreover, we observed that the heterogeneity of some morphological variable pairs such as *D* and *L*, *D* and *S*_*v*_, *D* and *D*_*v*_, *L* and *S*_*v*_ was ‘coupled’ with each other for this tumor ensemble, as indicated by the significant (p < 0.05) positive correlation coefficients of 0.75, 0.89, 0.73 and 0.82, respectively (Fig. 5c). However, we did not observe significant correlations between any morphological and hemodynamic variable pair indicating that morphological heterogeneity and hemodynamic heterogeneity were likely ‘decoupled’ in these tumors.

### Intra-tumor heterogeneity results in the establishment of unique microvascular ‘niches’

**Figure 6:**
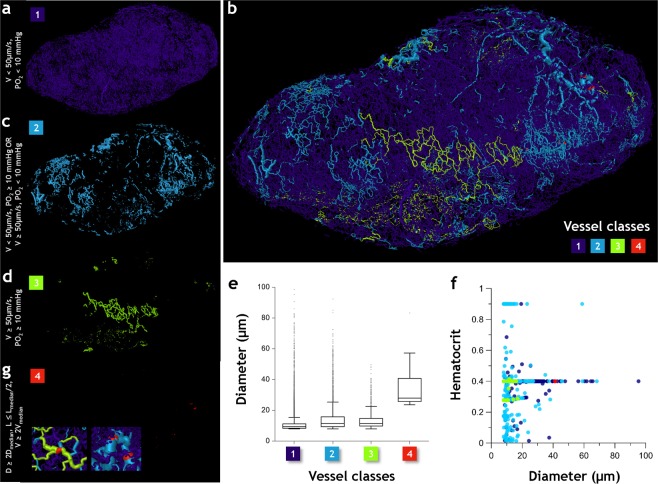
Intra-tumor heterogeneity results in the establishment of unique microvascular ‘niches’. Microvasculature in *Day 21* tumor (Tumor 1) is color coded into four classes based on their physiological characteristics and vascular structure. *Class 1* vessels (purple) (**a**) were hypoperfused and hypoxic (i.e. *Vel* < 50 µm/s and *PO*_*2*_ < 10 mmHg); *Class 2* vessels (light blue) (**c**) were hyperperfused and normoxic (i.e. *Vel* ≥ 50 µm/s and PO_2_ ≥ 10 mmHg); *Class 3* vessels (light green) (**d**) were either hypoperfused and normoxic or hyperperfused and hypoxic, and finally *Class 4* vessels (red) (**g**) served as functional ‘shunts’ (i.e. velocity at least 2× larger than the median blood flow velocity, vessel diameter at least 2× greater than the median diameter, and vascular length smaller than the median length by a factor of 0.5). Since occurrence of *Class 4* vessels is infrequent, the inset shows zoomed images illustrating their role as functional shunts within the tumor. (**b**) Spatial arrangement of these vessel classes in 3D. (**e**) Box and whisker plots of diameter (μm) for each vessel class, (**f**) plot of hematocrit vs. diameter (μm) for plasma-bearing vessels in each class.

Our simulations demonstrated that the heterogeneity of the tumor microvasculature and resulting hemodynamics combined to establish unique microvascular niches within the tumor. Figure 6 illustrates the four tumor vessel classes identified based on tumor hemodynamic definitions in the extant literature^31,34,35^. *Class 1* vessels (Fig. 6a) were hypoperfused and hypoxic (i.e. blood flow velocity, Vel < 50 µm/s and PO_2_ < 10 mmHg); *Class 2* vessels (Fig. 6c) were hyperperfused and normoxic (i.e. Vel ≥ 50 µm/s and PO_2_ ≥ 10 mmHg); *Class 3* vessels (Fig. 6d) were either hypoperfused and normoxic (i.e. low velocity and normal oxygenation) or hyperperfused and hypoxic (i.e. normal velocity but low oxygenation). Collectively, *Class 3* vessels lack the full functionality of *Class 2* vessels, yet are capable of maintaining transport better than *Class 1* vessels. Finally *Class 4* vessels (Fig. 6g) served as functional ‘shunts’ (i.e. velocity at least 2× larger than the median blood flow velocity, vessel diameter at least 2× greater than the median diameter, and vascular length smaller than the median length by a factor of 0.5) within the tumor. Figure 6b illustrates how one can visualize this heterogeneous hemodynamic landscape in Tumor 1 by mapping each vessel class onto the underlying microvascular network. Almost 90% of the vascular volume in this tumor was accounted for by *Class 1* vessels that were poorly or minimally perfused, hypoxic and also exhibited the smallest median diameter among all vascular classes (Fig. 6e**)**. Functionally active vessels of *Class 2* exhibited the greatest variation in hematocrit distribution (Fig. 6f), and were localized to the tumor rim along with *Class 3* vessels. *Class 4* vessels created short pathways of low resistance that diverted blood flow between vessel *Classes 1* and *2*, and *Classes* 2 and 3 (Fig. 6b,g).

### Ensemble-based multiparametric data classify the emergent angiogenic landscape better than either morphological or hemodynamic parameters alone

**Figure 7:**
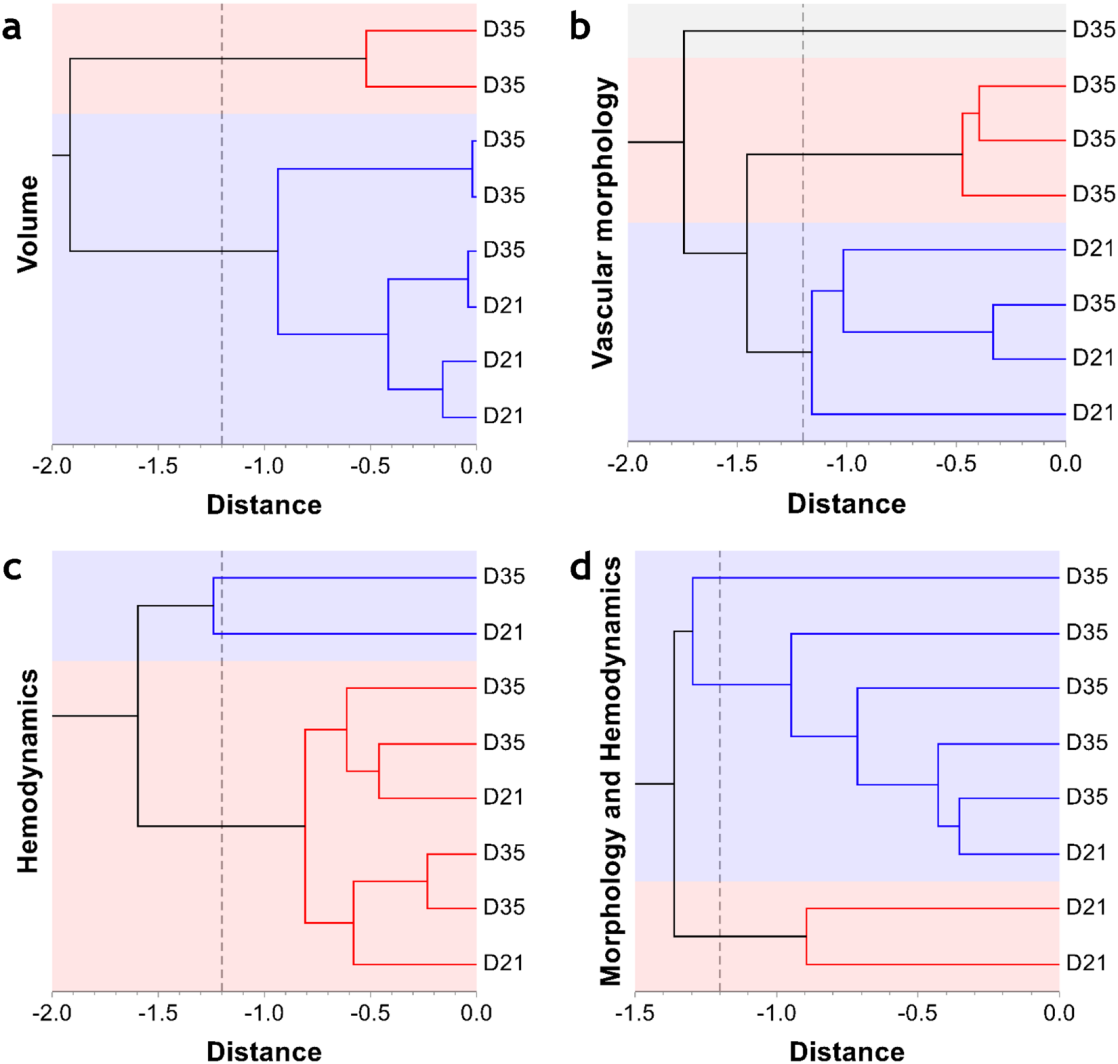
Whole-tumor ensemble derived multiparametric data classify the emergent angiogenic landscape better than either morphological or hemodynamic parameters alone. Hierarchical clustering of tumors as D21 (*Day 21* tumors) or Day35 (*Day 35* tumors) based on the unweighted pair-group method (hatched vertical line indicates the distance threshold). Automatic stratification of tumors into D21 and D35 categories based on: (**a**) tumor volume, (**b**) select vascular morphology parameters (*L*, *L*_*v*_, *S*_*v*_, *D*_*v*_), (**c**) select hemodynamic parameters (*Q*, *V*el, PO_2_, *vstt*, *MPL*), and (**d**) a combination of select vascular morphology and hemodynamic parameters (*L*, *L*_*v*_, *S*_*v*_, *D*_*v*_, *vstt* and PO_2_). Cophenetic correlation coefficients for these clusters were 0.88 0.82, 0.85 and 0.84, respectively. The misclassified D35 sample is Tumor 4 (Fig. 1d) and the misclassified D21 sample is Tumor 3 (Fig. 1c). It should be noted that one *Day 35* tumor (Fig. 1d) exhibited morphological features that strongly overlapped with that of one *Day 21* tumor (Fig. 1c) and was thus repeatedly found in the cluster corresponding to the *Day 21* tumors.

Finally, we utilized our rich multiparametric, morphological and hemodynamic data to classify the emergent angiogenic phenotype as belonging to *D21* (i.e. *Day 21* group) or *D35* (i.e. *Day 35* group). Figure 7a demonstrates that employing a traditional metric such as tumor volume alone was insufficient for classifying the tumor phenotype when hierarchical clustering was used. Figure 7c illustrates how heterogeneity of the hemodynamic parameters precluded such classification. In contrast, Fig. 7b,d illustrate how either the morphological parameters alone, or the morphological and functional variables taken together could classify the emergent angiogenic phenotype more efficiently. It should be noted that one *Day 21* tumor (Fig. 1d) exhibited morphological features that overlapped with that of one *Day 35* tumor (Fig. 1c), and was thus classified in the cluster corresponding to the *Day 35* tumors.

## Discussion

Tumor microenvironmental heterogeneity is emerging as one of the most important determinants of drug resistance and therapeutic response in cancer patients^3^. Therefore, to identify more effective drug regimens, a comprehensive characterization of tumor heterogeneity is being sought in the clinic via the collection of multiple biopsy samples from the same patient before and after therapy^1^. While this is a promising start, the search for a non-invasive approach that can be used safely in a large number of patients has resulted in the development of image-based methods for quantifying tumor heterogeneity^36^. Such ‘radiomic’ approaches include techniques that mine heterogeneity ‘features’ from large repositories of clinical imaging data for stratifying patients for therapy^37^. Other image-based approaches to investigate the tumor microenvironment include *in silico* simulations that incorporate imaging data as inputs to mathematical models^38^. The advantage of such computational approaches is that they permit the simultaneous investigation of multiple microenvironmental variables^38,39^. Since access to patient biopsy samples is often limited, high-fidelity imaging data from preclinical xenograft models can play a crucial role in elucidating tumor heterogeneity, especially when used as inputs to image-based computational models of cancer biology. For example, we recently demonstrated the feasibility of using high-resolution, whole-tumor micro-CT data for modeling the blood flow distribution in a whole-tumor microvascular network^40^. However, assessing emergent inter- and intra-tumor microenvironmental heterogeneity requires that we employ 3D data from multiple whole-tumors and interrogate multiple microenvironmental parameters. To achieve this, we developed an image ensemble-based computational and visualization framework that employed data from a collection of tumor xenografts to model the heterogeneity of microenvironmental variables such as vascular morphology, hemodynamics and intravascular oxygenation.

Our tumor-ensemble approach provides a novel method for mapping and quantifying inter- and intra-tumor heterogeneity in whole-tumor microvascular networks. This approach enabled the quantitative visualization of spatial heterogeneity of key vascular morphological parameters at high spatial resolution. We successfully demonstrated how spatial patterns of intra-tumoral heterogeneity could be exploited to identify differences in the vascular phenotype of tumors. For example, we discovered that advanced *Day 35* tumors were characterized by large inter-vessel distances, small vascular lengths and surface area densities. These trends were consistent with our current knowledge about tumor progression and the morphological changes that accompany breast cancer angiogenesis^41^. During the highly dysregulated process of tumor angiogenesis, rampant cell proliferation leads to a disproportionate increase in tumor volume relative to functional vessel sprouting, which in turn results in large avascular areas within the tumor such as those observed in our spatial heterogeneity analysis. Moreover, quantifying the distance to nearest vessel provided information on the length scales associated with extravascular diffusion in these irregular microvascular networks. Our 3D distance maps revealed areas of the tumor where effective delivery of drugs and nutrients could prove challenging due to the large extravascular diffusion distances^26^.

A significant consequence of our image-based computational framework was that it provided a solution to the long-standing technical hurdle of simulating blood flow and intravascular oxygenation in solid tumors using the whole-tumor 3D microvasculature. The profound inter- and intra-tumor heterogeneity of tumor vascular function we observed from our tumor ensemble simulations was consistent with the heterogeneity in blood flow and oxygenation measured in multiple solid tumor samples by other groups^29,31,32,42^. The distributions of blood flow rates and intravascular oxygenation were positively skewed^42,43^, indicating that a large sub-population of tumor vessels was inadequately perfused and under-oxygenated. Perfused vessels within each tumor exhibited high and intermediate oxygen tensions, but the median simulated oxygenation matched microelectrode-based measurements of intravascular oxygen tension in mammary tumors^32,42^ as well as other tumors^29,33^ (see Supplementary Tables S1 and S2). While the majority of the simulated blood flow values were within the ranges reported in the literature, we observed that a small fraction (<0.5% of the total vessels) of perfused vessel segments in each tumor exhibited high blood flow velocities (>10 mm/s). This may have resulted from the heterogeneous reconstructed vascular network of tumors or small anomalies in the 3D reconstruction of the microvascular topology from our μCT imaging data. Mapping intravascular oxygenation heterogeneity in whole-tumor networks enabled the visualization of the spatial distribution of perfusion-limited hypoxic vessels, which in combination with diffusion-limited hypoxic regions are known to be major drivers of breast tumor progression and metastasis^44,45^. Moreover, our indices of intra-tumoral hemodynamic heterogeneity could potentially be used to explore how these variables correlate with the probability of metastatic dissemination as well as with treatment outcome^46,47^. A powerful aspect of this image-based computational approach was that our hemodynamic simulations recapitulated emergent properties of the whole-tumor. For example, we were able to demonstrate how heterogeneity of the tumor microvasculature gave rise to unique microvascular niches that comprised of tumor vessels with distinct morphological and hemodynamic phenotypes. Therefore, one can foresee how image-based simulations of these intra-tumoral habitats could be combined with complementary genomic and metabolomics data to better understand the impact of tumor heterogeneity in breast cancer patients^48,49^.

Assessing inter- and intra-tumor heterogeneity using a tumor-ensemble revealed how vascular morphological heterogeneity resulted a heterogeneous functional phenotype. This analysis allowed us to gain critical insights into the emergent angiogenic phenotype. We illustrated how morphological and hemodynamic variables could collectively classify the emergent angiogenic phenotype better than hemodynamic variables alone. Therefore, it might be worth developing a systematic ensemble-based multiparametric heterogeneity index for tumor phenotyping rather than utilizing traditional metrics such as tumor volume^37^. Realistic simulations of angiogenic heterogeneity using the *de facto* tumor vasculature also have the potential to aide in the identification and development of non-invasive clinical biomarkers of tumor angiogenesis^50^. Collectively, our findings demonstrate the potential of combining structural and functional imaging biomarkers to help characterize the angiogenic status of a tumor or when quantifying its response to antiangiogenic therapies^51,52^.

Despite the advantages of this high-fidelity image-based computational framework, our approach did have some inherent limitations. For example, although we reconstructed more than 90% of vascular network nodes for all tumors to account for incomplete vascular filling of microvessels, we were unable to assess the effect of the remaining 10% of the nodes. Studies using percolation-based network models have shown that a significant fraction (>40%) of vessels needs to be excluded from the microvascular network before any observable impact on network connectivity or simulated blood flow distribution^53^. Since our aim was to model intravascular oxygenation for an entire tumor ensemble, we used a simplified model of oxygen transport^54^. The computational cost of this approach per simulation session was ~10 days on a local workstation (2.8 GHz, quad-core with 8 GB RAM) running parallelized MATLAB code. This simulation run-time could be dramatically reduced by switching to GPU-accelerated computing, an area we are currently exploring.

Since it is challenging to perform 3D reaction-diffusion-convection simulations for the whole-tumor volume, the current formulation for oxygen transport did not include the 3D extravascular diffusion of oxygen in the tissue. Although intratumoral blood flow can be influenced by other factors such as physical stress and the interaction between tumor cells and stromal components^55^, the current computational algorithm did not incorporate this diversity in cellular composition. We have utilized tumor data from two imaging time points (*Day 21* and *Day 35*), however, our computational framework can also be used to study earlier stage tumors or tumors following therapy, as long as each tumor exhibits a patent microvascular network that that can be perfused with a contrast agent that is visible using 3D imaging approaches (e.g. MRM, μCT or multiphoton microscopy). Finally, the current blood flow simulations were conducted for steady-state conditions and therefore do not recapitulate the temporal aspects of hemodynamic heterogeneity known to exist within tumors^14,56^, which is an ongoing area of future development.

It is worth mentioning that our computational approach was based on tumor microvasculature derived from an orthotopic xenograft model of human breast cancer. Since most tumor xenografts only proliferate in immune compromised animals, their microenvironments lack the immune components exhibited by human tumors. Additionally, tumor xenografts do not recapitulate all the genetic diversity observed in primary human tumors^57^. Due to these limitations, our analysis of tumor vascular heterogeneity may not recapitulate every aspect of phenotypic heterogeneity observed in breast cancer patients. However, that was not the primary goal of the current study and our approach did recapitulate salient features of the breast tumor microenvironment such as hypoxia, physiologically anomalous blood flow and the emergence of distinct microvascular niches^3^. We expect future computational applications to be based on images derived from syngeneic tumor models or patient derived xenografts (PDX) in immunocompetent animals. A potential avenue for computationally modeling cancer heterogeneity in patients would be to employ vascular data directly derived from *in vivo* breast cancer angiography or resected breast cancer tissue in which the vasculature has been labeled by some means. This approach seems feasible owing to recent advances in optical clearing methods in conjunction with novel optical imaging techniques such as light-sheet microscopy. For example, recently a fixed human brain tissue sample (3 × 3 × 1 cm^3^) was optically cleared to acquire high-resolution images of the brain microvasculature^58^. We are hopeful that these techniques would soon be available for optical clearing of patient derived tumor tissue.

The image-based platform we developed here would be ideal for preclinical investigations of drug development applications, such as studying vascular phenomena at the whole-tumor scale including vessel adaptation and remodeling^56^ in response to antiangiogenic therapies, and other treatments that target the vasculature^55^. Multiparametric datasets generated using this platform could also be useful for future clinical investigations. For example, when high-resolution measurements of tumor vasculature in patients become clinically available using *in vivo* CT angiography^59^, personalized simulations could predict the tumor’s angiogenic status and drug distribution levels. Physician-researcher partnerships^49^ could facilitate such efforts, since not all clinical settings may have the state-of-the-art computational resources necessary for simulating tumor hemodynamics. As a potential clinical application of this approach, *in silico* phenotypic data of metastatic breast cancer patients could be combined with their genetic and metabolic profiles to develop patient-specific data-libraries^49^ that could be shared widely and be used for predicting metastatic risk or stratifying patient treatment.

In conclusion, we believe that the development of an integrated imaging and computational platform based on high-fidelity tumor ensemble data will help establish: (i) a freely downloadable, multimodality atlas for cancer systems biology investigators and novel *in silico* applications; (ii) a computational model of antiangiogenic resistance that facilitates discovery of translational biomarkers for identifying patients who would benefit most from antiangiogenic agents; (iii) an *in silico* platform for testing novel therapeutics that can circumvent antiangiogenic resistance in breast cancer. We expect this image-based computational framework to be adaptable to other vasculature-dependent diseases such as stroke^60^ and myocardial infarction^61^.

## Methods

### Imaging of the 3D tumor ensemble

According to the experimental protocol^20^, 3 × 10^6^ triple-negative human breast cancer (MDA-MB-231) cells were inoculated orthotopically into the mammary fat pad of eight female Ncr-nu/nu mice. The animals were perfused with Microfil (FlowTech Inc., Carver, MA) a radio-opaque vascular filling agent, on *Day 21* or *35* post inoculation. *Ex vivo* μCT imaging was performed at Numira Biosciences (Salt Lake City, UT) on a high-resolution (8 μm) scanner (μCT40, ScanCo Medical, Brüttisellen, CH). The following parameters were used: 55 kVp, 300 ms exposure time, 2000 views and 5 frames per view. Since tumors were perfusion-fixed prior to perfusing with Microfill and imaging was conducted *ex vivo*^20^, cellular damage from X-ray exposure was not a concern. *Ex vivo* MRM images were acquired on a vertical bore Bruker 9.4 T spectrometer using a 10 mm volume RF coil (Bruker BioSpin Corp, Billerica,MA) and a T2*w MGE sequence with the following parameters: TE = 4.9/9.7/14.5/19.3/24.0/28.8 ms, TR = 150 ms, θ = 30°, NA = 14, resolution = 40 × 40 × 40 μm^3^. 3D visualization of whole tumors (Fig. 1) was achieved by co-registering *ex vivo* μCT images with MRM images^20^ using Amira® (Visage Imaging, San Diego, CA, USA). All animal experiments were conducted in accordance with an approved Johns Hopkins University Animal Care and Use Committee (JHU ACUC) protocol. The Johns Hopkins University animal facility is accredited by the American Association for the Accreditation of Laboratory Animal Care and meets National Institute of Health standards as set forth in the “Guide for the Care and Use of Laboratory Animals”.

### Model for Tumor Perfusion

To perform hemodynamic simulations, we reconstructed the topology of whole-tumor microvascular networks from 3D µCT images using a bioinformatics-based algorithm we recently reported^40^. The one-dimensional blood flow model^11,40^ encompassed the generalized 1D Poiseuille law, mass conservation at vessel junctions and the nonlinear rheological effects of blood flow (i.e. Fahraeus, Fahraeus-Lindqvist and plasma skimming effects) that are known to be significant in the microcirculation^21,62^. An optimization algorithm, that was based in part on a prior study^63^, monitored the vessel segments that feed and drain blood from the network, and optimized the boundary pressures to ensure mass conservation. Using these optimized pressures as boundary conditions, a system of linear equations was solved to obtain pressure distribution at the vessel junctions^40^. For a microvascular network for which complete boundary data is available (e.g. the rat mesentery network used for model validation in this study), our blood flow model can be run without implementing the optimization algorithm.

### Model for Intravascular Oxygen Transport

We employed the oxygen model introduced by Goldman and Popel with appropriate boundary conditions to estimate intravascular oxygen distribution within each whole tumor^54^. The current implementation is a highly simplified model and does not consider the three-dimensional extravascular diffusion of oxygen in the tissue because it is very challenging to perform 3D reaction-diffusion-convection simulations for the whole-tumor volume. As an approximation, the tumor volume was divided into multiple tissue domains whose volumes are proportional to corresponding vessel segment lengths, *L*_*ij*_. The total flux from the vessel segment is then proportional to tumor oxygen consumption rate; we utilized the rates determined experimentally for breast cancer xenografts by Vaupel *et al*.^35^. Therefore, this approach is equivalent to a generalized Krogh Cylinder Model, but without the geometric representation; thus, the tissue oxygen distribution cannot be calculated from Krogh-type approximation.

The governing equations for the individual segments can be described as follows:

1 $${Q}_{{b}_{i,j}}\cdot ({\alpha }_{{b}_{i,j}}\cdot {\rm{\Delta }}{P}_{i,j}+{H}_{{D}_{i,j}}\cdot {C}_{bind}\cdot {\rm{\Delta }}{S}_{Hb})={J}_{wal{l}_{i,j}}$$

where,

2 $${J}_{wal{l}_{i,j}}={M}_{c}\cdot {m}_{tissue}\cdot \frac{{L}_{ij}}{{\sum }_{i,j=1}^{N}{L}_{ij}}$$

3 $${\alpha }_{{b}_{i,j}}={H}_{{T}_{i,j}}\cdot {\alpha }_{RBC}+(1-{H}_{{T}_{i,j}})\cdot {\alpha }_{pl}$$

4 $${S}_{H{b}_{i,j}}({P}_{i,j})=\frac{{P}_{ij}^{{n}_{hill}}}{{P}_{ij}^{{n}_{hill}}+{P}_{50}^{{n}_{hill}}}$$

Here, values of blood flow ${Q}_{{b}_{ij}}$, discharge hematocrit ${H}_{{D}_{ij}}$ and tube hematocrit ${H}_{{T}_{ij}}$ in the individual vessel segments *ij* of length *L*_*ij*_ (where *i* and *j* represent branching or boundary nodes) are estimated from the blood flow model. *P*_*ij*_ is the oxygen tension in the blood; *C*_*bind*_ and ${S}_{H{b}_{ij}}$are red blood cells’ binding capacity and oxygen saturation respectively; ${J}_{wal{l}_{ij}}$is the total oxygen flux across the vessel wall for vessel segment of length *L*_*ij*_; *M*_*c*_ is the oxygen consumption rate for breast cancer xenografts derived from Vaupel *et al*.^35^, *m*_*tissue*_ is the tissue mass; *n*_*hill*_ is the Hill coefficient for hemoglobin binding cooperativity (2.7)^64^, *P*_50_ is the constant for 50% hemoglobin oxygen saturation (37 mmHg)^64^ and *α*_*pl*_ is the oxygen solubility in plasma (2.82 × 10^−5^ ml O_2_/ml tissue/mmHg)^64^.

Equation 1, with application of appropriate boundary conditions, can be transformed to the following system of equations:

5 $$({D}_{int}+{S}_{int})\cdot {P}_{int}=({D}_{bound}+{S}_{bound})\cdot {P}_{bound}+{J}_{wall}$$

where, *D*_*int*_ and *S*_*int*_ are the sparse matrices containing the transport coefficients for dissolved and bound oxygen derived from Equation 1 for branching nodes; *P*_*int*_ is the oxygen tension vector for branching nodes; *D*_*bound*_ and *S*_*bound*_ are the vectors of intravascular transport coefficients for boundary segments; *P*_*bound*_ is the oxygen partial pressure vector for boundary segments. This system is solved for oxygen tension (*P*_*int*_) at all branching nodes and then *S*_*Hb*_ values are updated based on Equation 4. The process is iterated until convergence.

Since the oxygen flux per unit length $({J}_{wal{l}_{ij}}/{L}_{ij})$ is assumed to be constant across each vessel wall segment (Equation 2), this term was incorporated only in the calculations for the vessels that transport significant amounts of oxygen subject to following limitations: discharge hematocrit, *H*_*D*_ > 0.01 and vascular segment transit time, *vstt* < 25 s. In a more stringent approach, the local flux per unit length is determined by matching the intravascular and extravascular transport^64^; nevertheless, the approximation of a constant flux used here provides useful indices of intravascular oxygenation.

### Validation of the blood transport model

To validate the predictions from our image-based computational framework, we employed a widely used and publicly available dataset derived from a 546 segment rat mesentery microvascular network^21^. We implemented our blood transport model for this public dataset as described in Pries *et al*.^65^. The boundary conditions for these simulations consisted of measured blood flow and discharge hematocrit values at all inlets (n = 31), and measured blood flow values at all outlets (n = 5), except the main venular outlet node that was maintained at constant pressure^21^. In addition, we compared the results of hemodynamic simulations based on our tumor ensemble data with experimental and simulated data from the extant literature^21,27–29,31,33^.

### 3D visualization of heterogeneous tumor vascular morphology, function and vessel classes

To quantify heterogeneity of the tumor vascular morphology, we generated 3D high-resolution (8 × 8 × 8 µm^3^ voxel) maps of distance to nearest blood vessel from the reconstructed whole-tumor microvasculature images in Amira (Visage Imaging, San Diego, CA, USA) using the Distance Map Module. The value assigned to each voxel was its 3D Euclidian distance to nearest blood vessel. We generated 3D maps of vascular length density and surface area density from the reconstructed skeletonized vasculature using custom Matlab code (MathWorks, Inc.). First, every vascular pixel was assigned an effective vascular length (and surface area) based on its position along the vessel. Next, the resulting data was convolved with a 20 × 20 × 20 kernel to estimate average vascular length density, *L*_*v*_ (mm/mm^3^) or surface area density, *S*_*v*_ (mm^2^/mm^3^) in a tumor sub-volume *V*_*ROI*_ = 160 × 160 × 160 µm^3^. To render 3D visualizations of spatial distributions of blood flow and intravascular oxygenation, we used the spatial graph visualization module in Amira® wherein each vessel was scaled by its mean diameter and color-coded by the functional parameter being displayed.

We set thresholds for blood flow velocity (50 µm/s) and oxygenation (10 mmHg) based on tumor hemodynamics definitions in the extant literature^31,35^ to identify the first three tumor vessel classes. While vessels exhibiting velocity smaller than 50 µm/s and oxygenation less than 10 mmHg were grouped into *Class 1*, *Class 3* vessels were those that demonstrated greater velocities and higher oxygenation than these thresholds. *Class* 2 vessels represented the class of vessels that lacked the full functionality of *Class 3* vessels, but were still capable of maintaining transport better than *Class 1* vessels. Finally, *Class 4* vessels represented functional shunts^34^ that exhibited velocity at least 2× greater than the median blood flow velocity, vessel diameter at least 2× greater than the median diameter, and vascular length smaller than the median length by a factor of 0.5. To map the spatial distribution of each class in 3D space, we used the spatial graph visualization module in Amira wherein each vessel was scaled by its mean diameter and color-coded by its class.

### Calculation of heterogeneity indices

Substantial variations in tumor vascular morphology and hemodynamics make it challenging to compare the heterogeneity of one vascular parameter with another. Therefore, to quantify inter- and intra-tumor heterogeneity within the tumor ensemble, we estimated the coefficient of variation (*CV*) (i.e. the ratio of the standard deviation to the mean) for each parameter distribution as a normalized measure of its heterogeneity. To determine the strength of the association between the heterogeneity of different parameters, we computed the Pearson correlation coefficient between every parameter pair.

### Statistical Analyses

Two-tailed Mann-Whitney U test (α = 0.05) was performed using the NCSS statistical software (NCSS, Kaysville UT) to determine whether there was a significant difference between the two tumor groups (i.e. *Day 21* and *Day 35*) in terms of median morphological properties such as distance to nearest vessel, vascular length and diameter, as well as median hemodynamic properties such as vascular segment transit time and oxygenation. To ensure valid clustering results, Cophenetic correlation coefficients were computed using the NCSS statistics package (NCSS, Kaysville UT) and values greater than 0.8 deemed significant for hierarchical clustering.

### Calculation of vascular parameters for hierarchical clustering

To classify the emergent angiogenic phenotype as belonging to *D21* (i.e. *Day 21* group) or *D35* (i.e. *Day 35* group), we implemented the unweighted pair-group method of hierarchical clustering in NCSS on the median values of vascular parameters. These included tumor volume, *Vol* (mm^3^); vascular morphological parameters such as length, *L* (mm); vascular length density, *L*_*v*_ (mm/mm^3^); surface area density, *S*_*v*_ (mm^2^/mm^3^); distance to nearest vessel *D*_*v*_ (µm); and the following vascular functional parameters: blood flow rate, *Q* (ml/s), flow velocity, *u* (mm/s); wall shear stress, *τ* (dyne/cm^2^); and vascular segment transit time, *vstt* (s)^40^, and intravascular oxygen tension, *PO*_2_ (mmHg)

6 $${u}_{ij}=\frac{4\cdot {Q}_{ij}}{\pi \cdot {D}_{ij}^{2}}$$

7 $${\tau }_{ij}=\frac{32\cdot {\mu }_{ij}\cdot {Q}_{ij}}{\pi \cdot {D}_{ij}^{3}}$$

8 $$vst{t}_{ij}=\frac{\pi \cdot {D}_{ij}^{2}\cdot {L}_{ij}}{4\cdot {Q}_{ij}}$$

here, *D*_*ij*_ is the segment diameter; *μ*_*ij*_ is the segment apparent viscosity; and *Q*_*ij*_ is the simulated segment blood flow rate. The mean path length, *MPL* (µm) for each tumor was computed as:

9 $$MPL=\frac{{\sum }_{i,\,j=1}^{N}{Q}_{ij}\cdot {L}_{ij}}{{\sum }_{i,\,j=1}^{N}{Q}_{ij}}$$

## Supplementary information

Supplementary Material

## Data Availability

All data supporting the findings of this study are available within the article and its supplementary information files. The Matlab code for all modules of the image-based computational pipeline presented in this study and associated 3D data are available from the corresponding author upon reasonable request.
