# Supplementary material for: Tumor Ensemble-Based Modeling and Visualization of Emergent Angiogenic Heterogeneity in Breast Cancer
Source: Sci Rep. 2019 Mar 27;9:5276. doi: 10.1038/s41598-019-40888-w (PMC6437174; doi:10.1038/s41598-019-40888-w)
Supplement: Supplementary file 1 — Supplementary Material [file 41598_2019_40888_MOESM1_ESM.pdf]

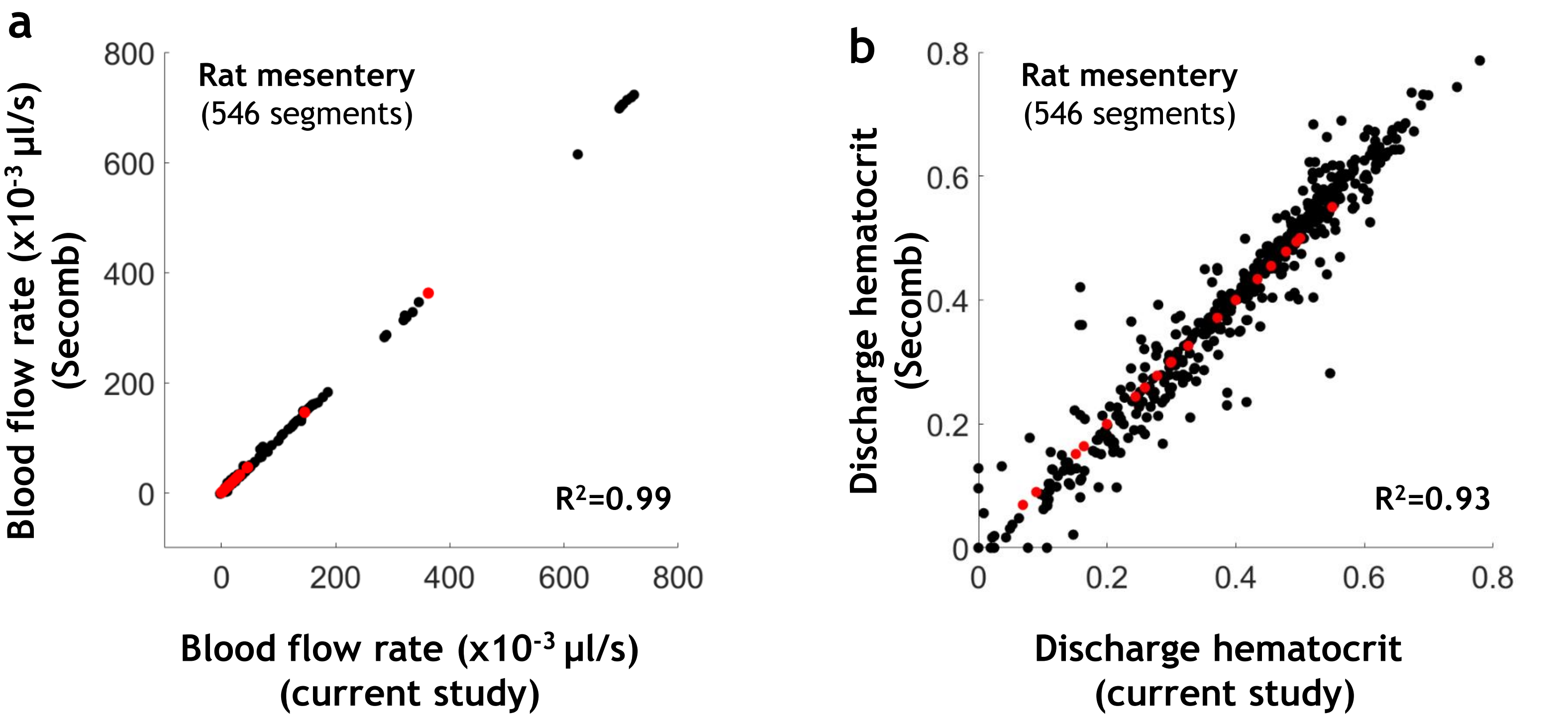

Supplementary Figure S1

Supplementary Table 1

Vascular parameters<br>Tumor ensemble	Current study<br>Mean	Literature<br>(solid tumors)<br>Mean ± SD
Vascular length density (mm/mm^3)	34.5	131 ± 84.7<br>[22, 23, 25]
Vascular surface area density (mm^2/mm^3)	22.9	15 ± 14<br>[22, 23, 25]
Distance to the nearest vessel (μm)	35	120 ± 132<br>[24, 26]
Blood flow rate (ml/s)	0.37×10^{-6}	$36 \pm 150 \times 10^{-6}$<br>[28, 29]
Velocity (mm/s)	0.9	0.85 ± 1.45<br>[27, 28, 29, 31]
Intravascular Oxygenation (mmHg)	13.4	14.4 ± 15.8<br>[29, 30, 32, 33]

22 Forster, J. C., Harriss-Phillips, W. M., Douglass, M. J. & Bezak, E. A review of the development of tumor vasculature and its effects on the tumor microenvironment. *Hypoxia* **5**, 21-32, (2017).

23 Hilmas, D. E. & Gillette, E. L. Morphometric analyses of the microvasculature of tumors during growth and after x-irradiation. *Cancer* **33**, 103-110, (1974).

24 Konerding, M. A. *et al*. Evidence for characteristic vascular patterns in solid tumours: quantitative studies using corrosion casts. *Brit J Cancer* **80**, 724-732, (1999).

25 Vogel, A. W. Intratumoral Vascular Changes with Increased Size of a Mammary Adenocarcinoma: New Method and Results. *Journal of the National Cancer Institute* **34**, 571-578, (1965).

26 Baish, J. W. *et al.* Scaling rules for diffusive drug delivery in tumor and normal tissues. *Proc Natl Acad Sci U S A* **108**, 1799-1803, (2011).

27 Brizel, D. M. *et al.* A Comparison of Tumor and Normal Tissue Microvascular Hematocrits and Red-Cell Fluxes in a Rat Window Chamber Model. *Int J Radiat Oncol* **25**, 269-276, (1993).

28 Leunig, M. *et al.* Angiogenesis, Microvascular Architecture, Microhemodynamics, and Interstitial Fluid Pressure during Early Growth of Human Adenocarcinoma Ls174t in Scid Mice. *Cancer Res* **52**, 6553-6560, (1992).

29 Torres, I. P., Leunig, M., Yuan, F., Intaglietta, M. & Jain, R. K. Noninvasive Measurement of Microvascular and Interstitial Oxygen Profiles in a Human Tumor in Scid Mice. *P Natl Acad Sci USA* **91**, 2081-2085, (1994).

30 Wilson, D. F. & Cerniglia, G. J. Localization of Tumors and Evaluation of Their State of Oxygenation by Phosphorescence Imaging. *Cancer Res* **52**, 3988-3993, (1992).

31 Kamoun, W. S. *et al.* Simultaneous measurement of RBC velocity, flux, hematocrit and shear rate in vascular networks. *Nat Methods* **7**, 655-U678, (2010).

32 Dewhirst, M. W. *et al.* Perivascular Oxygen-Tensions in a Transplantable Mammary-Tumor Growing in a Dorsal Flap Window Chamber. *Radiat Res* **130**, 171-182, (1992).

33 Vaupel, P., Kallinowski, F. & Okunieff, P. Blood-Flow, Oxygen and Nutrient Supply, and Metabolic Microenvironment of Human-Tumors - a Review. *Cancer Res* **49**, 6449-6465, (1989).

Supplementary Table S1

Supplementary Table 2

Tumor	Number of perfused vessels	**Vessel diameter (μm)** Median, IQR [28, 66]	**Vessel length (μm)** Median, IQR [22]	**Blood flow rate ($\times 10^{-6}$ μl/s)** Median, IQR [28,33]	**Velocity (mm/s)** Median, IQR [27, 28, 31, 32]	**Oxygenation (mmHg)** Median, IQR [29,30,32,33]
1	7883	11.9, 6.4	64.2, 61.1	5.7 ,92.2	0.04, 0.7	10.3, 10.2
2	5418	19.3, 14.4	73, 81.7	81.4, 320.9	0.32, 1.1	4.7, 10.4
3	2849	16.3, 13	68.5, 70.7	19.1, 109.1	0.09, 0.6	15.2, 16.4
4	2685	16.7, 11.6	77.6, 87.5	7.7, 45.5	0.04, 0.23	17.2, 10.9
5	1998	17.1, 12.5	89.7, 154.3	24.2, 132.2	0.1, 0.6	16.3, 9
6	5151	14.7, 11.8	132.1, 194.5	100.8, 280.3	0.6, 1.9	12.9, 6.9
7	7316	19.8, 13.7	72.3, 80.2	52, 317	0.2, 1.1	17.8, 10.2
8	3554	15.7, 12.6	72.6, 89.7	5.1, 32.5	0.02, 0.9	18.1, 16.6

22 Forster, J. C., Harriss-Phillips, W. M., Douglass, M. J. & Bezak, E. A review of the development of tumor vasculature and its effects on the tumor microenvironment. *Hypoxia* **5**, 21-32, (2017).

27 Brizel, D. M. *et al.* A Comparison of Tumor and Normal Tissue Microvascular Hematocrits and Red-Cell Fluxes in a Rat Window Chamber Model. *Int J Radiat Oncol* **25**, 269-276, (1993).

28 Leunig, M. *et al.* Angiogenesis, Microvascular Architecture, Microhemodynamics, and Interstitial Fluid Pressure during Early Growth of Human Adenocarcinoma Ls174t in Scid Mice. *Cancer Res* **52**, 6553-6560, (1992).

29 Torres, I. P., Leunig, M., Yuan, F., Intaglietta, M. & Jain, R. K. Noninvasive Measurement of Microvascular and Interstitial Oxygen Profiles in a Human Tumor in Scid Mice. *P Natl Acad Sci USA* **91**, 2081-2085, (1994).

30 Wilson, D. F. & Cerniglia, G. J. Localization of Tumors and Evaluation of Their State of Oxygenation by Phosphorescence Imaging. *Cancer Res* **52**, 3988-3993, (1992)

31 Kamoun, W. S. *et al.* Simultaneous measurement of RBC velocity, flux, hematocrit and shear rate in vascular networks. *Nat Methods* **7**, 655-U678, (2010).

32 Dewhirst, M. W. *et al.* Perivascular Oxygen-Tensions in a Transplantable Mammary-Tumor Growing in a Dorsal Flap Window Chamber. *Radiat Res* **130**, 171-182, (1992).

33 Vaupel, P., Kallinowski, F. & Okunieff, P. Blood-Flow, Oxygen and Nutrient Supply, and Metabolic Microenvironment of Human-Tumors - a Review. *Cancer Res* **49**, 6449-6465, (1989).

66 Hashizume, H. et al. Openings between defective endothelial cells explain tumor vessel leakiness. *Am J Pathol* **156**, 1363-1380, (2000).

Supplementary Table S2
